# Determinants of Work-Related Musculoskeletal Disorders Among Food Delivery Riders in Eastern Peninsular Malaysia: An Ergonomic Risk Assessment

**DOI:** 10.3390/healthcare13060645

**Published:** 2025-03-15

**Authors:** Aziah Daud, Ijlal Syamim Mohd Basri, Elyas Ahmad, Suhaily Mohd Hairon, Nor Azali Azmir, Azlis Sani Md Jalil, Rusli Nordin

**Affiliations:** 1 Department of Community Medicine, School of Medical Sciences, University Sains Malaysia, Kota Bharu 16150, Kelantan, Malaysia; ijlalsyamim@student.usm.my (I.S.M.B.); elyasahmad@student.usm.my (E.A.); suhailymh@usm.my (S.M.H.); 2 Faculty of Mechanical & Manufacturing Engineering, Universiti Tun Hussein Onn, Parit Raja 86400, Johor, Malaysia; azali@uthm.edu.my (N.A.A.); azlis@uthm.edu.my (A.S.M.J.); 3 Faculty of Medicine, Bioscience and Nursing, MAHSA University, Jenjarom 42610, Selangor, Malaysia; rusli@mahsa.edu.my

**Keywords:** work-related musculoskeletal disorders, WMSD, determinants food delivery rider, Eastern Peninsular Malaysia

## Abstract

**Background/Objectives:** Work-related musculoskeletal disorders (WMSDs) have been found to be the leading factor in disabilities and absenteeism among workers. Despite their growing numbers, WMSD prevalence and risk factors among food delivery riders remain underexplored. Given the high WMSD prevalence among motorcyclists and the rising road traffic accidents (RTAs) among delivery riders in Malaysia, a cross-sectional study was conducted to identify the determinants of WMSDs among this neglected group. **Methods**: A self-reported questionnaire consisting of sociodemographic factors, work-related factors, and a validated Malay-translated Standardised Nordic Musculoskeletal Questionnaire (M-SNMQ) was used to evaluate the WMSD symptoms and related factors among 191 food delivery riders in Eastern Peninsular Malaysia. An ergonomic risk assessment using the Rapid Entire Body Assessment (REBA) method and whole-body vibration (WBV) measurement was also conducted to elicit the WMSD risk and vibration exposure among the riders. The determinants of WMSDs were analysed using multiple logistic regression using SPSS 20.4. **Results**: This study revealed a high prevalence of WMSDs (74.9%) among food delivery riders in Eastern Peninsular Malaysia. Furthermore, three factors were found to be significantly associated with WMSDs among riders, namely the increasing average working days (aOR = 2.00; 95% CI = 1.34, 2.98; *p* = 0.001), whole-body vibration (WBV) above the exposure action value (EAV) limit (aOR = 2.71; 95% CI = 1.13, 6.53; *p* = 0.026), and not exercising before work (aOR = 21.63; 95% CI = 7.45, 62.79; *p* < 0.001). **Conclusions**: Targeted interventions are essential to mitigate ergonomic risks and enhance rider safety. Occupational health policies should prioritise pre-work exercise and WBV exposure reduction to minimise musculoskeletal strain. Future longitudinal studies are needed to evaluate the long-term impact of these risks on riders’ health.

## 1. Introduction

The fourth industrial revolution (IR4.0) has brought about changes in work trends, decreased job task redundancy, improved the skills of respected workers and machinery, and significantly lowered the cost of production [1]. In the food and beverage industry, by using a booking application, consumers can easily purchase food from restaurants, which cuts down the waiting time. In fact, with the advancement of technology, consumers can have their meals from their preferred restaurants sent to them by food delivery riders. In Malaysia, delivery services have consistently risen in popularity among Malaysians and become one of the most sought-after services, especially during the COVID-19 pandemic. The COVID-19 pandemic significantly impacted the food and beverage industry, especially during the early phase of the pandemic, when many restrictions were imposed on this sector. For example, dining at restaurants was not allowed, and only vaccinated citizens were permitted to visit stores. As a result, food delivery services emerged as a crucial tool for the public. The pandemic also caused many people to lose their jobs. As the government of Malaysia categorised food delivery as an “essential service” during a series of Movement Control Orders (MCOs), working as a food delivery rider became one of the options available [2]. According to Bernama [3], more Malaysians joined food delivery services as riders during the COVID-19 pandemic due to the loss of their previous jobs and the high demand for food delivery services during that time. However, with the exponential increase in the number of people becoming food delivery riders in Malaysia, the incidence of road traffic accidents (RTAs) among riders also keeps rising, with a high mortality rate; for instance, 1700 mortality cases were recorded among food delivery riders in 2021 compared to 115 cases in 2019 [4].

Studies suggest that WMSDs may contribute to road traffic accidents (RTAs) among motorcyclists by affecting their physical endurance, reaction time, and riding posture, although the exact causal mechanisms remain complex and multifactorial. According to Michael et al. [5], WMSDs can lead to psychological distress such as anxiety, depression, and stress among riders. Riders with psychological distress frequently exhibit inappropriate riding habits, such as speeding, which ultimately result in RTAs. Work-related musculoskeletal disorders (WMSDs) are highly discussed among health practitioners today. WMSDs include any musculoskeletal diseases that are related to work or aggravated by work, which usually affect the back area, the lower limbs, and the upper limbs [6]. They are a very common problem among industrial workers and can affect their health and productivity. In the long run, WMSDs indirectly cause disruption in the global economy. In the United States alone, WMSDs were among the major contributor to the economic burden, where the estimated loss of private wages and salaries among construction workers was more than USD 45 million in 2014. In particular, back injuries accounted for more than 40% of WMSDs among construction workers in the United States from 1992 to 2014 [7]. In many studies across the globe, WMSDs have been proven to cause more absenteeism and disabilities among workers than other diseases [8,9,10].

In Malaysia, WMSDs were the highest occupational disease reported to the Social Security Organisation (SOCSO) in 2019. On top of that, more than 30% of the reported WMSD cases were compensated [11]. Various studies regarding WMSDs in various fields have been performed in Malaysia, and the prevalence of WMSDs was significantly high in almost all studies. For example, the prevalence of WMSDs among male traffic policemen using high-powered motorcycles was 67.9% [12]. Furthermore, the prevalence of WMSDs among workers in shipyard industries was more than 70%, and almost 60% of palm oil workers reported neck pain [13,14]. A preliminary study by Samad et al. [15] revealed a high prevalence of back pain (56%) among 39 food delivery riders in the Kota Bharu district in Kelantan, Malaysia. While extensive research has been conducted on WMSDs among industrial workers and office employees, studies focusing on food delivery riders remain scarce. Existing studies often do not account for the unique occupational conditions of delivery riders, such as prolonged motorcycle use, exposure to vibration, and the high variability in working hours. However, the reported WMSD cases among workers, especially riders, keep increasing over time, which necessitates conducting more studies on WMSDs. Therefore, research gaps still exist in many areas regarding WMSDs, which need to be addressed to reduce the burden of WMSDs among riders.

The World Health Organisation (WHO) concluded that the causes of WMSD development among workers are multifactorial [6]. The cause can originate from humans themselves or the environment. Food delivery riders often experience prolonged riding durations, frequent acceleration–deceleration cycles, and exposure to environmental factors such as heat and rain. These conditions contribute to increased musculoskeletal stress and a higher likelihood of WMSDs. The nature of delivery work also limits opportunities for posture adjustment, leading to sustained static postures and increased ergonomic risk. Previous studies have identified several factors to be associated with the development of WMSDs such as sociodemographic factors, environmental factors, and work-related factors, such as awkward posture, vibration exposure, and working duration. In many studies, WMSD occurrences have been strongly associated with female workers. Among Brazilian adults, female workers were two times more likely to develop WMSDs than male workers [16]. Furthermore, many studies revealed body mass index (BMI) to be one of the factors associated with WMSDs [17,18]. In addition, many studies have revealed an association between muscle stretching and WMSDs. It was found that stretching before starting work helps to reduce the risk of developing WMSDs [19]. Moreover, awkward posture while working was found to be highly associated with WMSDs among workers. A study among female workers in a hazelnut factory in Nigeria revealed that unergonomic neck posture was among the factors associated with the high prevalence of neck pain [20]. In the past decade, more ergonomic-based studies have been carried out involving workers to establish the best ergonomic posture for workers [21,22]. Ergonomics among workers have been improved in many sectors through technological advancements. For example, engineering innovation in designing shock absorber tools among mechanics in Indonesia helps to improve the Rapid Entire Body Assessment (REBA) score from 13 to 2, where a lower REBA score indicates a lower risk of developing WMSDs [23].

Aside from environmental and occupational factors, long working hours also contribute to a high prevalence of WMSDs among workers. Many studies have proven that long working hours are significantly associated with the development of WMSDs among workers [24,25,26]. In addition, long working hours are often associated with prolonged exposure to maintained/static postures and physical inactivity, which may contribute to the development of WMSDs. For those in heavy industries and transportation, chronic exposure to vibration has been proven to be associated with the development of WMSDs among workers, as indicated in various studies across the world. In the United States, occupational exposure to whole-body vibration (WBV) and hand–arm vibration (HAV) were significantly associated with the development of WMSDs in the neck and shoulder region [27]. The same conclusion was also elucidated in a study among metropolitan bus drivers in India by Hanumegowda and Gnanasekaran [28]. A reduction in WBV exposure among workers was shown to reduce the risk of developing WMSDs. According to the European Vibration Protection Directive 2022/44/EC, WBV exposure, expressed as daily vibration exposure A(8), should not exceed the exposure action value (EAV) limit, which is 0.5 m/s^2^. In addition, employers are required to take action and implement control measures once the A(8) exceeds the EAV level because the risk of developing WMSDs will be high [29].

This study aimed to explore the magnitude of the problem and elicit the factors associated with the development of WMSDs among food delivery riders in Eastern Peninsular Malaysia. Understanding these factors is crucial for developing targeted interventions and workplace policies to reduce WMSD prevalence in this vulnerable workforce. Specifically, we hypothesise that long working hours, whole-body vibration exposure, and a lack of pre-work exercise are significantly associated with WMSDs among food delivery riders.

## 2. Materials and Methods

### 2.1. Study Site

This study was conducted in Eastern Peninsular Malaysia. This region is bordered in the northwest by Kelantan, in the southwest by Pahang, and in the east by the South China Sea. The estimated population in 2021 was 1.1 million with a Malay majority. In 2021, more than 1000 registered food delivery riders were registered with more than 5 delivery service companies in this region, making the region the preferred study site. The ergonomic posture assessment and whole-body vibration (WBV) measurement were conducted in outdoor locations where food delivery riders commonly gather, such as designated rider waiting areas and rest stops, to ensure minimal disruption to their work schedules. These assessments were performed by three trained professionals with expertise in occupational health and ergonomics.

### 2.2. Study Design and Recruitment Method

A cross-sectional study was conducted among 191 food delivery riders in Eastern Peninsular Malaysia. The sample size was calculated using a single proportion formula by taking 20% dropout into consideration using G*Power software version 3.1.9.7. The study criteria included all registered food delivery riders who had worked for more than six months. Additionally, riders with congenital musculoskeletal disorders were excluded from this study. This study employed a snowball sampling method for participant recruitment. The initial recruitment began with a “Captain Rider”, an experienced food delivery rider who leads a network of other riders within the region. A pre-survey interview with the Captain Rider was conducted to understand the best approach for reaching potential participants while ensuring their privacy and confidentiality. The Captain Rider then assisted in identifying and inviting eligible food delivery riders who met the study criteria. After agreeing to participate, these riders further referred their peers, expanding the sample pool in a chain-referral manner. This method was particularly effective as many riders were hesitant to disclose their identity due to their previous employment status before joining the food delivery industry. A total of 230 food delivery riders were initially approached, and 191 met the inclusion criteria and agreed to participate. The final sample size was determined based on feasibility and logistical considerations, ensuring adequate statistical power for the analysis. All respondents were provided with detailed study information, and informed consent was obtained before data collection. The recruitment process continued until the required sample size was achieved. In addition, written consent was given prior to the study.

### 2.3. Data Collection Methods

This study consisted of three main parts: a self-reported questionnaire, an ergonomic posture assessment using the Rapid Entire Body Assessment (REBA) method, and whole-body vibration (WBV) measurement.

#### 2.3.1. Self-Reported Questionnaire

In this study, data regarding WMSDs were collected using a self-reported Malay-translated Standardised Nordic Musculoskeletal Questionnaire (M-SNMQ), validated by Amin et al. [30]. The original version of the Standardised Nordic Musculoskeletal Questionnaire was developed by Kuorinka et al. [31] and is used in various occupational fields. The M-SNMQ has demonstrated strong test–retest reliability, with kappa agreement values of at least 0.75, making it a reliable tool for assessing WMSD symptoms. No modifications were made, ensuring its reliability and suitability for the food delivery rider population. The questionnaire consisted of four sections, namely sociodemographic factors, work-related characteristics, motorcycle factors, and WMSD assessment using the M-SNMQ. Sociodemographic factors, work-related characteristics, and motorcycle factors were selected based on their association with the development of WMSDs in previous studies. In the sociodemographic section, the respondents answered questions regarding age, gender, race, marital status, education status, height (cm), weight (kg), and smoking status. Body mass index (BMI) was calculated later using the formula weight (kg)/height (cm)^2^. Height and weight were measured using a standardised stadiometer and digital weighing scale. The same set of calibrated instruments was used for all participants, and all measurements were conducted by trained researchers to ensure consistency. Meanwhile, in the section on work-related characteristics, the respondents were asked to answer questions regarding their working experiences, average trips per day, average working hours per day, average working days per week, and any exercise or muscle stretching performed at least five minutes before work. The non-validated, work-related factors questionnaire was designed by the authors based on relevant occupational conditions observed among food delivery riders to ensure it accurately captured the nature of their work environment and routine. Regarding motorcycle use, the respondents were asked to answer several questions pertaining to their motorcycles, including the type of motorcycle used, manufacturer, engine power, motorcycle age, service frequency, and suspension service.

The M-SNMQ was used to evaluate WMSD symptoms among the riders, and it was designed according to eight anatomical locations, namely the neck, shoulders, upper back, lower back, arms, thighs, knees, and feet. At the end of the session, those who complained of WMSD symptoms at any anatomical site were classified as having WMSDs, while those who did not have any symptoms in the past 12 months were classified as not having WMSDs. The whole questionnaire was designed to be completed within 30 min. The respondents completed the questionnaire in their free time without disturbing their daily job.

#### 2.3.2. Ergonomic Posture Assessment Using REBA Method

**Figure 1:**
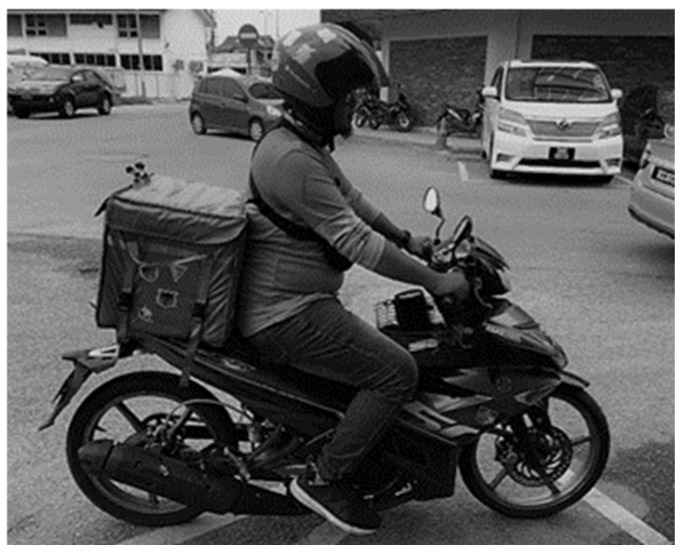
The working posture of a food delivery rider.

This postural analysis was developed by Hignett and McAtamney [32]. It was chosen due to its practicality and reliability. Moreover, REBA provides a comprehensive assessment of the ergonomic risk factors associated with a specific task or job. Furthermore, it has high inter-rater and intra-rater reliability in postural risk assessment. It assesses the entire body, including the upper and lower limbs, the trunk, and the neck, and assesses the interaction between body segments and task demands. The REBA method was applied to all 191 riders to determine the postural risk of developing WMSDs. This postural assessment method had a specified scoring system involving almost all anatomical human structures, such as the neck, trunk, leg, upper arm, lower arm, and wrist. In addition to the postural analysis, the REBA method was used to assess other WMSD risk factors, such as load handling, coupling, repetitive movements, and sudden postural changes. The final REBA score was then compared with the WMSD risk table. To capture the working posture of the riders, the frozen video recording method was used when the riders started to ride their motorcycles to deliver food to customers. As riders have limited capacity to adjust their posture and usually maintain the same position throughout the riding process, the working posture was taken at only one point in time, which was at the beginning of their journey to deliver food to customers, as shown in Figure 1. The working posture was analysed and scored using the REBA tool worksheet. The final step was to compare each rider’s final REBA score to the WMSD risk table. The WMSD risk table consists of the final REBA score, the level of WMSD risk, and the action level with respect to the urgency for control measures, where a higher final REBA score indicates a higher risk of developing WMSDs, which corresponds to a higher urgency of intervention, thus indicating that changes should be made at the workplace.

#### 2.3.3. Whole-Body Vibration Measurement

The WBV measurement was conducted using a calibrated Larson Davis HVM 100 Human Vibration Meter (PCB Piezotronics, Inc., Provo, UT, USA) equipped with a tri-axial accelerometer seat pad, in compliance with ISO 2631 standards [33]. The calibration was carried out by technical specialists at Universiti Tun Hussein Onn Malaysia (UTHM) prior to data collection to ensure measurement accuracy. The WBV measurement was carried out when riders had their orders ready to be delivered to their clients in order to avoid interference with their trip and income. The measuring time began when they left to deliver food to customers and ended when they returned to the starting location (one round trip). The data obtained from the HVM were transferred into the “Blaze Software V6.2.3”. WBV was measured in accordance with ISO 2631 guidelines with regard to the anatomical positioning and the direction of the accelerometer. The average cumulative vibration exposure from the x, y, and z axes was calculated and converted into an 8 h weighted value. WBV was expressed as daily vibration exposure A(8). At the end of the session, the daily vibration exposure A(8) was compared with the daily vibration exposure A(8) limit values, which are EAV (0.500–1.149 m/s^2^), and the exposure limit value (ELV) (≥1.15 m/s^2^), as regulated by the EU Directive 2002/44/EC [29]. Finally, WBV exposure was categorised into two groups, depending on whether the daily vibration exposure A(8) exceeded the exposure action value (EAV) or not.

### 2.4. Statistical Analysis

In this study, IBM SPSS version 20.4 was used to analyse the data. All continuous data were presented using the mean (SD), while categorical data were presented using frequencies and percentages. To determine the factors associated with WMSDs among the riders, a univariable analysis was carried out using simple logistic regression, including all of the sociodemographic factors and occupational factors, the REBA score, and WBV exposure. All variables that had *p*-values < 0.25 and were considered to be important were further tested using a multiple logistic regression test. The preliminary final model was obtained after conducting variable selection using the forward likelihood ratio (forward LR) selection and the backward likelihood ratio (backward LR) elimination methods. There was no significant possible two-way intervariable interaction in this study. The variance inflation factor (VIF) was found to be less than 10 in, which indicated that there was no multicollinearity present in the preliminary final model. The Hosmer–Lemeshow test, a classification table, and the area under the receiver operating characteristic (ROC) curve were used to assess the model fitness. For this study, the Hosmer–Lemeshow test was statistically non-significant (*p* = 0.240). The classification table showed a value of 86.4%, and the area under the ROC curve was 77.8%, with *p* < 0.001, which indicated that the model was well fitted.

### 2.5. Ethical Consideration

Ethical clearance for this research was obtained from the Human Research Ethics Committee of Universiti Sains Malaysia (JEPeM Code: USM/JEPeM/21030230). Confidentiality of the data was strictly prioritised.

## 3. Results

A total of 191 food delivery riders were recruited in this study, and the analysis was carried out accordingly. The sociodemographic characteristics, work-related characteristics, and motorcycle characteristics and WMSDs, REBA, and WBV measurements were evaluated descriptively, while the associated factors were evaluated using the odds ratio.

### 3.1. Sociodemographic Characteristics

Table 1 summarises the sociodemographic characteristics of the respondents selected in this study. In this study, most of the respondents (92.7%) were male. However, female respondents had a higher prevalence of WMSDs than male respondents. All riders were of Malay ethnicity, with a mean (SD) age of 27.6 (5.76) years old. Most of them were unmarried, but the prevalence of WMSDs was highest in the divorced/widowed category. In addition, most of them had a formal education, while only five of them had a non-formal education. Furthermore, the majority of the respondents (53.9%) were active smokers; however, an almost equal prevalence of WMSDs was observed among smokers and non-smokers. The mean (SD) height was 166.1 (14.08) cm, while the mean (SD) weight was 73.2 (20.62) kg. The calculated mean (SD) BMI based on height and weight was 26.01 (6.54).

### 3.2. Work-Related Characteristics

In this study, the respondents were selected among those who worked for more than six months as food delivery riders. Among them, about 52% had already worked for more than 1 year, while 48% had work experience of 6 to 12 months, with an almost equal prevalence of WMSDs in both groups. In addition, the average number of trips per day was 19 trips. Furthermore, the respondents had a mean (SD) number of working hours per day of 10.2 (2.33) hours. Meanwhile, they worked for 6 days a week on average. Most of them (83.8%) claimed that they had not performed any type of exercise or muscle stretching before beginning to work, as shown in Table 2.

### 3.3. Motorcycle Characteristics

Most of the respondents (83.8%) used sedan motorcycles for delivering food to customers. Meanwhile, 15.7% used scooters, and only one respondent used a high-powered motorcycle. The prevalence of WMSDs among riders was almost the same for sedan and scooter users, but the high-powered motorcycle user also had a WMSD. There were a variety of motorcycle manufacturers involved, and the most common (50.3%) motorcycle manufacturer was from the “C” company. In total, the motorcycles used by the riders were built by seven different manufacturers. In addition, the average motorcycle age used was 8.1 years. Furthermore, most of the riders (67%) had their motorcycle serviced on time, while 33% did not follow a service schedule. Meanwhile, the majority of them (73.3%) claimed they had never serviced the suspension of their motorcycles since buying them. Cross-tabulation showed a high prevalence of WMSDs (78.6%) among those who did not service their motorcycle’s suspension. The motorcycle characteristics are summarised in Table 3.

### 3.4. Prevalence of WMSDs

The prevalence of WMSDs among the riders is summarised in Table 4. This study revealed that the prevalence of WMSDs among food delivery riders in Eastern Peninsular Malaysia was high, where 74.9% complained of WMSD symptoms in one or more body regions for the past 12 months. Specifically, the back was the commonest body region the respondents noted having WMSD symptoms. In particular, lower back pain (LPB) was the WMSD symptom with the most (73.3%) complaints in the past 12 months. However, the least affected body regions with WMSD symptoms in the past 12 months were the feet and thighs, with prevalence rates of 3.1% and 2.6%, respectively.

### 3.5. Ergonomic Posture Assessment Using REBA

Table 5 summarises the final REBA scores among the respondents. The final REBA scores among the respondents were limited to scores of “4”, ”5”, and “6” with percentages of 36.1%, 31.4%, and 31.9%, respectively. However, there was one rider who had a final REBA score of 9. On average, the final REBA score was “5”.

### 3.6. WBV Measurement

In this study, most of the respondents (69.6%) had WBV exposure between 0.500 m/s^2^ and 1.149 m/s^2^. Meanwhile, three of the riders had WBV exposure of more than 1.149 m/s^2^, while the rest had WBV exposure of less than 0.500 m/s^2^, as shown in Table 6. Furthermore, the mean daily vibration exposure A(8) experienced by the riders was 0.624 m/s^2^.

### 3.7. Factors Associated with WMSDs

This study revealed three factors that were significantly associated with the development of WMSDs among riders. The first factor was the average working day. In this study, it was found that with the addition of one average working day, the likelihood of developing WMSDs was two times higher. This study also revealed that riders with WBV exposure above the EAV limit had two times the odds of having WMSDs compared to riders with WBV exposure below the EAV limit. The most striking finding was in terms of the exercise factor. This study concluded that riders who did not exercise before starting their work had 21 times the odds of having WMSDs compared to those who exercised before work, as summarised in Table 7.

## 4. Discussion

This study revealed a high prevalence of WMSDs (74.9%) among food delivery riders in Eastern Peninsular Malaysia. Similar findings have been found in many other studies involving motorcyclists [12,34,35]. Exposure to prolonged static posture and vibration are among the major contributing factors that predispose occupational motorcyclists to WMSDs. This phenomenon should be an eye-opener for health authorities in Malaysia, especially occupational health professionals. With an increasing number of people becoming food delivery riders in Malaysia atpresent, a higher impact of WMSDs on economic growth is evident, as the high prevalence of WMSDs among workers has been proven to indirectly cause economic drawbacks due to absenteeism and a reduction in productivity [8,9].

In the present study, female respondents were found to have a higher prevalence of WMSDs than male respondents. The same finding was elicited by Alias et al. [36], where female motorcyclists had a higher total number of musculoskeletal discomfort symptoms than male motorcyclists. This might be due to the different physiological attributes of different genders, such as muscle mass, fat distribution, laxity of the tendon, and bone density [37], which make female riders more prone to developing WMSD symptoms than their male counterparts.

In addition, this study also revealed high final REBA scores among the riders, with the majority of them having a final REBA score between four and six, which implies a medium risk of developing WMSDs. One rider had a final REBA score of nine, which denotes a high risk of WMSDs. Based on the WMSD risk table, further investigation must be conducted, and changes must be implemented to improve the working posture of riders in order to reduce their risk of WMSDs. The same result was obtained by Montolalu et al. [38], who found a medium risk of WMSDs among online transportation drivers in Indonesia.

This study also proved several factors to be significantly associated with the development of WMSDs among food delivery riders in this region. Firstly, the riders with WBV above the EAV limit value had almost three times higher odds of having WMSDs than those with WBV below the EAV limit. Many studies have also suggested that WBV is among the risk factors of WMSDs among workers; for instance, in a study of back disorders among crane operators in the Netherlands by Bongers et al. [39], the authors concluded that the combination of WBV exposure, unfavourable posture, and climatic conditions was significantly associated with back disorders among crane operators. In 2002, the European Agency for Safety and Health at Work, through Directive 2002/44/EC, established a regulation according to which there must be a minimum basis of protection for workers to overcome the adverse effects of mechanical vibration on the musculoskeletal system of the human body. The directive resulted in guidelines on the limit values for daily vibration exposure, i.e., the exposure action limit (EAV) for WBV should not exceed 0.5 m/s^2^, and the exposure limit value (ELV) should not exceed 1.15 m/s^2^ [29]. However, the mean daily vibration exposure of the riders in this study was 0.624 m/s^2^, which exceeded the EAV limit value. This reflected the high prevalence of WMSDs among the riders in this study and aligned with the multiple regression results, where WBV above the EAV limit had a significant association with the development of WMSDs. In many studies, exposure to chronic vibration has been proven to cause significant disruption in human physiology, especially in the musculoskeletal system, where it can cause degeneration of the intervertebral disc and changes in muscle morphology [40,41].

This study also revealed that the riders who did not perform any type of exercise or muscle stretching before work had 21 times the odds of having WMSDs compared with those who performed muscle stretching before working. The same result was elicited in a study among nurses in China, where a lack of physical exercise was shown to be associated with the development of WMSDs among nurses [42]. Jepsen and Thomsen [43] also showed the effect of exercise in preventing upper limb symptoms among computer operators. In addition, some studies have proven the benefit of exercise for workers before starting their daily routine work, including an increase in muscle endurance and flexibility and a reduction in pain and disability [44,45]. Meanwhile, some other studies have shown that performing stretching exercises can reduce pain and improve range of motion (ROM) [46,47]. Both acute and long-term exercise and stretching have been proven to have benefits on human physiology, especially on the musculoskeletal system, as they can prevent the excessive accumulation of lactic acid in the muscle with the stimulation of group III muscle afferent fibres, which initiates the cascade to improve the muscle’s blood flow without using excessive muscle energy [48]. This will prevent muscle fatigue among workers, which will later result in the prevention of WMSD development. However, the high odds ratio for not exercising before work may be influenced by unmeasured confounders, such as overall physical activity levels. Future studies should explore this association with a more detailed assessment of exercise type and intensity.

The results also showed that the odds of having WMSDs among the riders were two times higher with the addition of one average working day. Longer working days reflect a longer duration of exposure to other risk factors of WMSDs, such as vibration dose and unfavourable static posture. In this study, working hours were not found to be significantly associated with WMSDs. This can be explained by the wide range of working days among the riders, which was from three days to seven days. So, even though the daily working hours were high, the cumulative working hours per week could be low if the riders worked only three days per week. To justify this explanation, it should be noted that many studies have proven that working hours are significantly associated with WMSDs when working days are constant [49,50]. Furthermore, the average cumulative working hours per week among the riders were also very high, at 60 h per week. According to the Employment (Amendment) Act 2022, the maximum working hours for workers should not exceed 45 h per week [51]. Thus, the high working hours per week explains the high prevalence of WMSDs among riders, as they are exposed to the risks of WMSDs for a longer duration. However, due to the lack of awareness and legal action among authorities, working hours are not being supervised thoroughly. Even worse, due to riders having the status of independent contractors, their working hours entirely depend on the riders themselves, as they can choose how long they wish to work.

### Strengths and Limitations

The main strength of this study was that it involved many areas, all of which can contribute to the development of WMSDs among food delivery riders, such as sociodemographics, working environments, ergonomics, and WBV exposure. The comprehensive tools used in this study, i.e., the M-SNMQ, REBA, and WBV measurement tools, are the features that differentiate this study from other studies, as this study encompassed most of the possible exposure risks for the development of WMSDs among food delivery riders.

Nevertheless, this study has limitations. There could be selection bias in this study due to the “healthy worker” effect (HWE). Thus, a higher prevalence of WMSDs should be anticipated by the related agencies. The high prevalence of WMSDs elicited in this study should serve as a warning to the related agencies, such as health authorities, to initiate related programmes to reduce the burden of WMSDs among riders. Furthermore, this study did not account for pre-existing health conditions, prior musculoskeletal injuries, or non-occupational activities that could influence WMSD prevalence. Future studies should include these factors to improve the robustness of their findings. In addition, WBV exposure was measured during one complete delivery trip to minimise participant burden and avoid disruptions to their work. However, this approach may not fully capture variations in road conditions. Future studies should consider extended measurement periods for a more comprehensive analysis.

## 5. Conclusions

In conclusion, this study proved that WBV above the EAV limit, not exercising before work, and long average working days were significant factors that contributed to the high prevalence of WMSDs among food delivery riders in Eastern Peninsular Malaysia. To reduce the magnitude of the problem, workers, employers, and the health sector must work closely to improve the working conditions for riders, especially regarding working hours. The maximum weekly working hours of 45 h must be imposed on riders to ensure they have enough rest before continuing their work. In addition, awareness programmes on the importance of daily exercise and stretching before starting work must be implemented to improve the quality of life among riders. Health authorities must therefore reconsider this matter in order to lessen the burden of WMSDs on riders, which will hopefully result in a future decrease in RTAs among riders. Furthermore, it is hoped that the results of this study will serve as a basis for other interventional studies involving many sectors to implement measures that can prevent motorcycle accident risks, such as improved ergonomics and vibration reduction, and therefore markedly reduce the prevalence of WMSDs among riders in the near future, thus improving the productivity and economic growth of this country.

## Figures and Tables

**Table 1:** Sociodemographic characteristics of food delivery riders in Eastern Peninsular Malaysia (*n* = 191).

Variables	*n* (%)	Mean (SD)	WMSD	
			Yes, *n* (%)	No, *n* (%)
Sex				
Male	177 (92.7)		131 (74.0)	46 (26.0)
Female	14 (7.3)		12 (85.7)	2 (14.3)
Age		27.6 (5.76)		
Race				
Malay	191 (100.0)			
Marital Status				
Unmarried	112 (58.6)		84 (75.0)	28 (25.0)
Married	72 (37.7)		53 (73.6)	19 (26.4)
Divorced/Widowed	7 (3.7)		6 (85.7)	1 (14.3)
Education				
Non-Formal	5 (2.6)		4 (80.0)	1 (20.0)
Primary School	4 (2.1)		2 (50.0)	2 50.0)
Secondary School	84 (44.0)		65 (77.4)	19 (22.6)
Form 6 */STAM **/Diploma	76 (39.8)		56 (73.7)	20 (26.3)
Degree/Master’s/PHD	22 (11.5)		16 (72.7)	6 (27.3)
Smoking				
Yes	103 (53.9)		68 (77.3)	20 (22.7)
No	88 (46.1)		75 (72.8)	28 (27.2)
Height (CM)		166.1 (14.08)		
Weight (KG)		73.2 (20.62)		
BMI		26.01 (6.54)		

* Form 6: Malaysian pre-university education equivalent to A-Levels. ** STAM: Sijil Tinggi Agama Malaysia (Higher Religious Certificate).

**Table 2:** Work-related characteristics of food delivery riders in Eastern Peninsular Malaysia (*n* = 191).

Variables	*n* (%)	Mean (SD)	WMSD	
			Yes, *n* (%)	No, *n* (%)
Work Experience				
6–12 Months	92 (48.2)		67 (72.8)	25 (27.2)
>12 Months	99 (51.8)		76 (76.8)	23 (23.2)
Average Trips/Day		18.9 (6.16)		
Working Hours/Day		10.2 (2.33)		
Average Working Days/Week		6.1 (1.03)		
Exercise Before Work				
No	160 (83.8)		137 (85.6)	23 (14.4)
Yes	31 (16.2)		6 (19.4)	25 (8.06)

**Table 3:** Motorcycle characteristics of food delivery riders in Eastern Peninsular Malaysia (*n* = 191).

Variables	*n* (%)	Mean (SD)	WMSD	
			Yes, *n* (%)	No, *n* (%)
Types of Motorcycle				
Sedan	160 (83.8)		120 (75.0)	40 (25.0)
Scooter	30 (15.7)		22 (73.3)	8 (26.7)
High-powered	1 (0.5)		1 (100.0)	0 (0.0)
Manufacturers				
A	79 (41.4)		58 (73.4)	21 (26.6)
B	2 (1.0)		2 (100.0)	0 (0.0)
C	96 (50.3)		72 (75.0)	24 (25.0)
D	1 (0.5)		0 (0.0)	1 (100.0)
E	2 (1.0)		1 (50.0)	1 (50.0)
F	2 (1.0)		2 (100.0)	0 (0.0)
G	9 (4.7)		8 (88.9)	1 (11.1)
Motorcycle Age (Year)		8.1 (6.24)		
Service Frequency				
Followed Schedule	128 (67.0)		94 (73.4)	34 (26.6)
Did Not Follow Schedule	63 (33.0)		49 (77.8)	14 (22.2)
Suspension Service				
Never	140 (73.3)		110 (78.6)	30 (21.4)
At Least Once	51 (26.7)		33 (64.7)	18 (35.3)

**Table 4:** The 12-month prevalence of WMSDs according to body regions (*n* = 191).

	Body Regions							
	Neck	Shoulders	Upper Back	Lower Back	Arms	Thighs	Knees	Feet
12-Month Prevalence, *n* (%)	31 (16.23)	35 (18.32)	106 (55.50)	140 (73.30)	10 (5.24)	5 (2.62)	11 (5.76)	6 (3.14)

**Table 5:** Final REBA scores (*n* = 191).

Frequency	Final REBA Score				Mean (SD)
	4	5	6	9	
*n* (%)	69 (36.1)	60 (31.4)	61 (31.9)	1 (0.5)	4.98 (0.88)

**Table 6:** Daily vibration exposure A(8) (m/s^2^) of food delivery riders in Eastern Peninsular Malaysia (*n* = 191).

Frequency	Daily Vibration Exposure (8 h Weighted) (m/s^2^)			Mean (SD)
	<0.500	0.500–1.149	≥1.150	
*n* (%)	55 (28.8)	133 (69.6)	3 (1.6)	0.624 (0.317)

**Table 7:** Multiple logistic regression * of factors associated with WMSDs among food delivery riders in Eastern Peninsular Malaysia (*n* = 191).

Variables	β	Wald Statistic (df)	Adjusted OR (95% CI)	*p*-Value
Average Working Days/Week	0.69	11.42	2.00 (1.34, 2.98)	0.001
WBV Above EAV				
No			1	
Yes	1.00	4.96 (1)	2.71 (1.13, 6.53)	0.026
Exercise				
Yes			1	
No	3.07	31.98 (1)	21.63 (7.45, 62.79)	<0.001

* Constant = −5.998; forward LR and backward LR methods were applied; no multicollinearity and no interaction between the variables; Hosmer–Lemeshow test, *p* = 0.240; classification table was 86.4% correctly classified; area under receiver operating characteristic (ROC) curve was 77.8%.

## Data Availability

The data presented in this study are only available on request from the corresponding author due to confidentiality and ethical restrictions.
